# Case Report: “Niemann-Pick Disease Type C in a Catatonic Patient Treated With Electroconvulsive Therapy”

**DOI:** 10.3389/fpsyt.2021.745734

**Published:** 2021-10-22

**Authors:** M. van Verseveld, L. H. Koens, Tom J. de Koning, R. L. E. Derikx, J. A. van Waarde

**Affiliations:** ^1^Department of Psychiatry, Rijnstate Hospital, Arnhem, Netherlands; ^2^Department of Neurology, University Medical Center Groningen, University of Groningen, Groningen, Netherlands; ^3^Expertise Center Movement Disorders Groningen, University Medical Center Groningen, Groningen, Netherlands; ^4^Department of Genetics, University Medical Center Groningen, University of Groningen, Groningen, Netherlands; ^5^Department of Pediatrics, Clinical Sciences, Lund University, Lund, Sweden

**Keywords:** electroconvulsive therapy, inborn errors of metabolism, Niemann-Pick type C, catatonia, seizure threshold

## Abstract

We describe a case of an adolescent male with Niemann-Pick Type C (NP-C), a neurodegenerative lysosomal lipid storage disorder, who presented with recurrent catatonia which required repeated treatment with electroconvulsive therapy (ECT). During the ECT-course, seizure threshold increased substantially, leading to questions about the influence of NP-C on neuronal excitability. In this exemplary ECT-patient, NP-C was diagnosed not until after the first ECT-course when initial psychopharmacology for catatonia had failed and antipsychotics and benzodiazepines showed significant *side-effects*. Clinicians should be aware of NP-C in patients referred for ECT, especially in the case of treatment resistance, neurological symptoms and intolerance of psychopharmacological drugs. As was shown in our NP-C patient, ECT can be repeatedly effective for catatonic features. In the literature, effectiveness of ECT in patients with NP-C has sparsely been reported. This case demonstrates that detection of NP-C is beneficial for patients because more optimal treatment with ECT can be provided earlier without further exposure to side-effects.

## Introduction

Niemann-Pick type C (NP-C) is a neurodegenerative lysosomal lipid storage disorder caused by mutations in the *NP-C1* or *NP-C2* gene. Onset of symptoms can range from infancy until adulthood, and symptoms can be very heterogeneous. In adult-onset NP-C, movement disorders and psychiatric symptoms are frequent (1, 2). Diagnosing NP-C in patients is often challenging and underdiagnosis is likely, particularly in patients with late-onset forms (3). Patients are frequently misdiagnosed with psychiatric disorders, including schizophrenia, atypical psychosis, and mood disorders (4, 5). Moreover, antipsychotic drugs may cause extrapyramidal side-effects more often in patients with NP-C. Otherwise, additional movement disorders caused by NP-C may be wrongly attributed to side-effects of antipsychotic drugs (6).

Early recognition of NP-C is important to provide the right counseling, diagnosis, and treatment. Diagnosing NP-C in an earlier stage will help both the patient and caregivers to better understand the condition, the diversity of (psychiatric) symptoms, and the possible treatments. NP-C disease modifying treatment with miglustat is available and needs to be considered (4–7). From the psychiatric perspective, treatment of psychiatric symptoms in patients with NP-C may differ from other psychiatric conditions. For example, use of typical antipsychotics in patients with NP-C needs to be avoided as these are more likely to cause extrapyramidal side-effects (6). Also, psychosis in these patients may appear treatment resistant, and antipsychotic drugs may cause paradoxical worsening (7, 8).

In general, electroconvulsive therapy (ECT) is effective and–therefore–a good alternative for patients suffering severe treatment-resistant psychosis, depression, catatonia, and delirium (9). ECT is safely performed under general anesthesia and with muscle relaxation. Using an electrical stimulus, neuronal functioning of the brain is altered. This elicits brief seizure activity which propagates throughout the brain and terminates by itself due to several inhibitory processes (10). In patients with NP-C, data about the effectiveness of ECT is limited. Also, the influence of abnormal lysosomal lipid storage, as in NP-C, on seizure initiation, propagation, and termination is unknown.

Here, we describe an adolescent male with NP-C who presented with catatonia and was successfully treated with ECT.

## Case Report

A 23-year-old male patient was referred to our center because of a second episode of catatonia. He had a medical history of major depressive disorder, delusions and catatonia when he was 16 years-of-age, but the patient was healthy otherwise. He was a successful secondary school pupil, loved playing piano, and worked as a volunteer. There was no family history of psychiatric disorders and no consanguinity. A psychologist estimated his intellect to be normal, with verbal and motor performance in the average range and the processing speed below average. During the first episode, the patients showed psychotic and catatonic symptoms. Underlying physical conditions were excluded by normal results of general laboratory and cerebral spinal fluid examinations (especially focusing on infections and paraneoplastic disorders), computed tomography (CT) and magnetic resonance image (MRI) of the brain. Treatment of the catatonic features with lorazepam was started (maximum 24 mg/24 h) without any improvement, and even showed a paradoxical worsening effect with disappearance of speed and functional physical activity. Subsequently, a course of ECT was initiated twice a week [bifrontotemporal (BL) electrode placement; initial seizure threshold (IST) 25.2 mC; electrical dose ranged: 50.4–151.1 mC, pulse width 0.5 ms, motor seizure duration ranged: 15–96 s, electroencephalography (EEG) seizure duration range: 22–257 s; device: Thymatron IV (Somatics Incorporation, Lake Bluff, Illinois, USA)], leading to prompt recovery after four ECT-sessions. ECT was then discontinued, against the ECT guideline. However, after eight days the patient showed a relapse of symptoms. Twenty-one more ECT-sessions followed, leading to complete remission of symptoms. Since bipolar disorder was among the working hypotheses, aripiprazole 30 mg was given as maintenance therapy. However, this was tapered off to stop after six months due to extrapyramidal side-effects.

After this ECT-course, the patient mentioned that he was no longer able to play piano, because he could not switch between the keys and the sheet music. Neurological examination revealed an inability to look up and in particular down, compatible with a vertical supranuclear gaze palsy (VSGP). Also, splenomegaly, subtle dystonia and parkinsonism were observed. This combination of psychiatric symptoms, movement disorders, and VSGP raised the suspicion of an underlying inborn error of metabolism. A diagnosis of NP-C was genetically confirmed [compound heterozygous mutations c.346C>T (p.R116X) and c.247A>G in the *NP-C1* gene]. Treatment with miglustat was initiated. After 14 months his treatment was terminated because of side-effects including headaches, lack of energy, cognitive impairment, and depressive mood. In the following weeks, after discontinuation of miglustat, his condition improved. After the discontinuation of the pharmacological therapy, the patient continued his homeopathic medicines, which he and his parents believe contributed to his improvement.

At 23 years of age he presented with a recurrence of catatonia. At admission, clouded consciousness, mutism, grimacing, stereotypy, mannerisms, negativism, excitement and perseveration were seen. He was rated with a severity score of 36 (out of 69) on the Bush-Francis Catatonia Rating Scale (BFCRS) (11). Laboratory examination, including whole blood count, electrolytes, renal function, vitamins, CRP, and liver panel showed no abnormalities. The lipid panel showed no abnormalities either, apart a low HDL 0.74 mmol/L (laboratory reference: >1.00 mmol). The cytochrome P450 genotyping showed increased CYP2D6 activity. MRI of the brain appeared normal. Treatment with oral lorazepam (maximum 7.5 mg/24 h) failed again and even showed a paradoxical worsening effect with psychotic features, disinhibition, and excitement. ECT was started with BL electrode placement, using a constant-current (0.9 ampère), brief-pulse (1.0 ms) device (Thymatron IV; Somatics Incorporation, Lake Bluff, Illinois, USA), with the first treatment dose at 75.6 mC based on his IST at the first ECT-course. Directly after the first ECT-session, improvement of the level of consciousness, mobility, and verbal responsiveness was observed and his condition progressed to full recovery after 11 BL ECT-sessions (ECT-doses ranged: 75.6–453.6 mC; pulse width 1.0 ms; motor seizure duration ranged: 49–85 s, EEG seizure duration ranged: 97–180 s). Remarkably, during this ECT-course the threshold for inducing seizures increased rapidly. After four ECT-sessions, a charge of 453.6 mC was needed to induce a seizure. After the ECT-course, the BFCRS score was rated zero. He was able to read, solve puzzles, and eventually ride his bicycle again. In accordance with the patient and his parents, no psychopharmacological maintenance treatment was prescribed because he had previously experienced severe side-effects. However, he continued the homeopathic medicines. One year later, his condition is still improving.

## Discussion

We present a case of a young patient showing recurrent catatonia, which was treated successfully with ECT repeatedly. In this exemplary ECT-patient, NP-C was diagnosed not until after the first ECT-course when initial psychopharmacologic treatment for catatonia had failed, and antipsychotics and benzodiazepines showed significant side-effects. Clinicians should be aware of NP-C in patients referred for ECT, especially in the case of treatment resistance, neurological symptoms (e.g., extrapyramidal signs), and intolerance of psychopharmacological drugs. During the ECT-course seizure threshold in this patient increased substantially, leading to questions about the influence of NP-C on neuroexcitability.

### Successful ECT in NP-C

Psychiatric symptoms are common in patients with late-onset forms of NP-C. In particular, NP-C patients may present with treatment resistant psychosis and mood disorders, which are indications for ECT (4–6). As was shown in our NP-C patient, ECT can be repeatedly effective for catatonic features. In the literature, effectiveness of ECT in patients with NP-C has sparsely been reported. Foels et al. describe successful ECT in catatonia in a patient with NP-C (12). Walterfang et al. present two patients with catatonic symptoms who were initially treated with ECT for schizophrenia, but who developed movement disorders, VSGP and cognitive decline afterwards, leading to the diagnosis of NP-C (13). Furthermore, successful treatment of psychosis and mood disorders with ECT is described in other subtypes of lysosomal storage disorders, including Tay-Sachs disease and adult neuronal ceroid lipofuscinosis (14). Based on these case reports, in patients with NP-C, ECT may be considered earlier in the treatment of psychosis, catatonia and mood disorders.

### Underdiagnosing NP-C in ECT-Patients

As was shown in our patient, NP-C was discovered later in the course of the disease, after he had received the first ECT-course. In general, it seems likely that among ECT-patients unidentified patients with NP-C can be found. Diagnosing NP-C is often challenging, particularly when patients present with psychiatric disorders. However, additional (neurological) symptoms like VSGP, movement disorders, cataplexy, and cognitive decline may aid to identify NP-C in those patients (1, 3). Also, treatment resistance, a paradoxical worsening effect, and extrapyramidal side-effects of antipsychotics may enhance the suspicion on NP-C (6, 7). For patients, detection of NP-C–as an important factor influencing treatment resistance and side-effects of psychopharmacological drugs (4, 13)–is important. ECT, as a more optimal treatment, can then be provided earlier without further exposure to side-effects (4).

For the clinician, there are several options for diagnosing NP-C, including molecular genetic analysis sequencing mutations in the *NP-C1* or *NP-C2* gene, the use of cholesterol esterification assays, and oxysterol assay-based screening (1, 2). Clinicians taking care of patients suffering treatment resistant psychosis, mood disorders, and catatonia may consider those diagnostic tools for NP-C more often. In clinical practice, examination of the eye movements for a gaze palsy may help to raise the suspicion of NP-C in order to refer to a physician specialized in inborn errors of metabolism (e.g., pediatrician, neurologist).

### NP-C and Neuroexcitability

ECT is often very effective when adequate seizure activity is elicited. Effects of ECT on neurogenesis, neuroendocrine dysregulation, and on molecular level of intracellular signaling are hypothesized, but the exact working mechanism of ECT is still unclear (15). Also, the influence of NP-C on the patient's brain processes during ECT-induced seizure activity is unknown, although the neuron is primarily affected in patients with NP-C. For instance, lipid accumulation, involving endocytosed cholesterol, occurs in neurons of the cerebral cortex, cerebellum, and hippocampus (2). This accumulation of endocytosed cholesterol and other abnormalities may lead to neuropsychiatric symptomatology (16, 17) but may also affect the brain's excitability. It may be hypothesized that there is an interaction between cholesterol, neuroexcitability, seizure activity, and the mechanisms of action of ECT. Accumulation of lipids, among which endocytosed cholesterol, in the neurons may potentially influence the seizure threshold and–therewith–possibly the effectiveness of ECT, because ECT is most optimal when the electrical stimulus exceeds the seizure threshold substantially (18). Interestingly, in our young NP-C patient, the seizure threshold increased rapidly during the ECT-course. This increase was not expected, because an increase in seizure threshold is associated with higher age (19). It may be assumed that this increase was (partly) due to a disturbed lipid, among which endocytosed cholesterol, storage in the neuron lowering the neuronal excitability in this NP-C patient. This hypothesis may be of interest for future studies.

To conclude, NP-C may be considered in ECT-patients who may present with (atypical) psychotic and catatonic features, treatment resistance, additional neurological symptoms, and severe side-effects from antipsychotics and benzodiazepines. Because these patients may be referred for ECT before a diagnosis of NP-C is considered, special attention of psychiatrists is warranted and referral to a physician specialized in inborn errors of metabolism needs to be considered. ECT may be very effective for treatment of catatonia in NP-C patients. Increase in seizure threshold during the ECT-course, even in young NP-C patients, may be seen. From a broader perspective, future studies may investigate the influence of in-brain cholesterol on neuroexcitability, seizure initiation, propagation, and termination in ECT.

## Data Availability Statement

The original contributions presented in the study are included in the article/supplementary material, further inquiries can be directed to the corresponding author.

## Ethics Statement

Ethical review and approval was not required for the study on human participants in accordance with the local legislation and institutional requirements. The patients/participants provided their written informed consent to participate in this study. Written informed consent was obtained from the individual(s) for the publication of any potentially identifiable images or data included in this article.

## Author Contributions

All authors contributed to the article and approved the submitted version.

## Conflict of Interest

The authors declare that the research was conducted in the absence of any commercial or financial relationships that could be construed as a potential conflict of interest.

## Publisher's Note

All claims expressed in this article are solely those of the authors and do not necessarily represent those of their affiliated organizations, or those of the publisher, the editors and the reviewers. Any product that may be evaluated in this article, or claim that may be made by its manufacturer, is not guaranteed or endorsed by the publisher.
